# An Estimate of Wolverine Density for the Canadian Province of Alberta

**DOI:** 10.1002/ece3.70702

**Published:** 2024-12-30

**Authors:** Jason T. Fisher, Mehnaz Jahid, Robin Gutsell, Anne Hubbs, Laura L. E. Cowen, Mirjam Barrueto, Nicole Heim, John Paczkowski

**Affiliations:** ^1^ School of Environmental Studies University of Victoria Victoria British Columbia Canada; ^2^ Department of Mathematics and Statistics University of Victoria Victoria British Columbia Canada; ^3^ Government of Alberta, Environment and Protected Areas, Edmonton and Rocky Mountain House Edmonton Alberta Canada; ^4^ University of Calgary Calgary Alberta Canada; ^5^ Yellowstone to Yukon Conservation Initiative Canmore Alberta Canada; ^6^ Government of Alberta, Forestry and Parks Canmore Alberta Canada

**Keywords:** boreal forest, conservation, *Gulo gulo*, mustelid, Rocky Mountains, species at risk

## Abstract

Wolverines ( *Gulo gulo* ) are a circumboreal species that has experienced substantial range reduction worldwide. In Canada, the wolverine has been extirpated entirely from the east, and from prairie regions in the west. The province of Alberta holds the south‐central portion of wolverines' Canadian range, and there they have been designated as *Data Deficient* since 2001 due to a historical lack of information. Our aim was to provide a first approximation of a wolverine abundance estimate at the provincial scale to inform science‐based management as well as status designation. We synthesised existing density estimates and wolverine–habitat relationships to create a province‐wide density estimate for wolverines. Densities were derived from five landscapes, spanning protected National Parks in the Rocky Mountains, the highly developed Foothills and the northcentral and northwestern boreal forests. Densities were estimated using spatially explicit capture–recapture (SECR) models. Densities ranged from 6.74 wolverines/1000 km^2^ in the northwest boreal to 0.71 wolverines/1000 km^2^ in the foothills. The proportion of adults was based on a study from the northwest, which estimated 57% adults to 43% subadults. Extrapolating densities across natural subregions (bioclimatic ecoregions), based on known habitat relationships, it was estimated that there were 955 wolverines in the province, of which 544 were adults. This number falls well below an IUCN threshold for a legally listed species; we suggest a reassessment of the wolverine status in Alberta and considering commensurate conservation actions.

## Graphical abstract

Wolverines live at their range limit in Alberta, Canada, and are subject to increasing pressures. We compiled multiple datasets across research studies to estimate wolverine density. We estimated much less than 1000 wolverine adults in the province, an IUCN threshold for a listed species, signalling the need for species assessment and potential conservation actions.

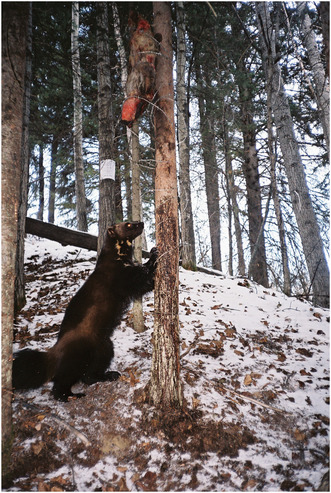

## 1 Introduction

Wolverines ( *Gulo gulo* ) are a circumboreal species that has experienced population declines in its Palearctic range (Bischof et al. 2020; Moqanaki et al. 2023) and substantial range reduction in its Nearctic range (COSEWIC 2014; Laliberte and Ripple 2004). In Canada, the wolverine has been extirpated from the east and prairie regions in the west (Cardinal 2004; COSEWIC 2014). The province of Alberta (661,849 km^2^) holds an estimated 5%–10% of wolverines' Canadian range. There they have been designated as *Data Deficient* since 2001 due to a historical lack of information on their population sizes, distribution, threats and ecology (Petersen 1997).

However, in the last two decades, a remarkable amount has been learned about wolverines globally, and in Alberta specifically (Fisher et al. 2022). Wolverines in and around the Rocky Mountains are highly sensitive to landscape development, especially the density of linear features such as petroleum exploration ‘seismic’ lines (Fisher et al. 2013; Heim et al. 2017). Throughout the Nearctic, extant wolverine distributions are associated with cold areas that retain spring snow (Copeland et al. 2010). Mechanisms posited include the snow‐den hypothesis wherein snow caves are needed for reproduction (Copeland et al. 2010) and the refrigeration‐zone hypothesis wherein cold‐caching food is required for cubs (Inman et al. 2012). Behavioural research suggests support for scatter hoarding cold caches that also defend against kleptoparasitism (van der Veen et al. 2020). The spring snow denning hypothesis has been challenged (Magoun et al. 2017; Persson et al. 2023); in Alberta, the spring snow pattern holds for Rocky Mountain wolverines (Heim et al. 2017) but not boreal wolverines (Webb et al. 2016). A third posited mechanism linked to kleptoparasitism is that snow mitigates competition for scavenging opportunities with more numerous anthrophilic coyotes ( *Canis latrans* ), with predictions supported by spatiotemporal co‐occurrence patterns (Chow‐Fraser et al. 2022; Heim et al. 2019); other mesocarnivores appear to play a similar competitive role (Bell et al. 2023).

Within the protected landscapes of the Rocky Mountain National Parks and adjacent public landbase to the west, wolverine populations have declined by 39% over the last decade due to trapping on provincial landbases, as well as snow and landscape change throughout (Barrueto et al. 2022; Barrueto, Sawaya, and Clevenger 2020; Mowat et al. 2020). In Alberta's boreal forest, telemetry data show wolverines avoid roadways for travel (roads are sources of collision mortalities) and variably use or avoid forest harvested areas (by sex and season) in ways different from the mountain landscapes (Scrafford et al. 2017, 2018; Scrafford and Boyce 2018).

Although knowledge of wolverine ecology has increased substantially in recent years, density throughout the Nearctic remains largely unknown, save for a few studied landscapes (Barrueto et al. 2022; Scrafford et al. 2024). Density—number of individuals in a given area—is an essential biodiversity variable (Pereira et al. 2013) and the basis of wildlife population models as it informs N, baseline population size (Williams, Nichols, and Conroy 2002). For managed wildlife species, it is from this N that natural mortality is deducted and reproduction added, to guide decisions about sustainable harvest. Without density, our harvest allocations become mere guesses (Williams, Nichols, and Conroy 2002), with overharvest a common consequence (Maxwell et al. 2016). Beyond harvesting decisions, density also informs conservation actions to manage other potential threats such as landscape management decisions and allowable human activity. Density and population size also form the basis for conservation listing, a trigger point for assigning status under the International Union for Conservation of Nature (IUCN) criteria (Commission 2001; Le Breton et al. 2019; Miller et al. 2007). Currently, there is no density estimate for Canada, or for any of its provincial or territorial jurisdictions, to guide IUCN status assessments or sustainable harvest decisions. As a consequence, those few well‐studied landscapes in Canada show local declines (Barrueto et al. 2022; Mowat et al. 2020).

Our overarching goal was to estimate wolverine densities for Alberta, Canada, to inform provincial status assessment and future harvest management and conservation decisions. Within this goal, our objectives were to (1) compile all data useful for informing wolverine densities in Alberta including boreal and mountain landscapes; (2) identify data that could be used to inform a provincial estimate; (3) propose a framework for extrapolating available density estimates across Alberta; and (4) provide recommendations for future research and monitoring.

## 2 Methods

### 2.1 Data Compilation and Assessment

**FIGURE 1:**
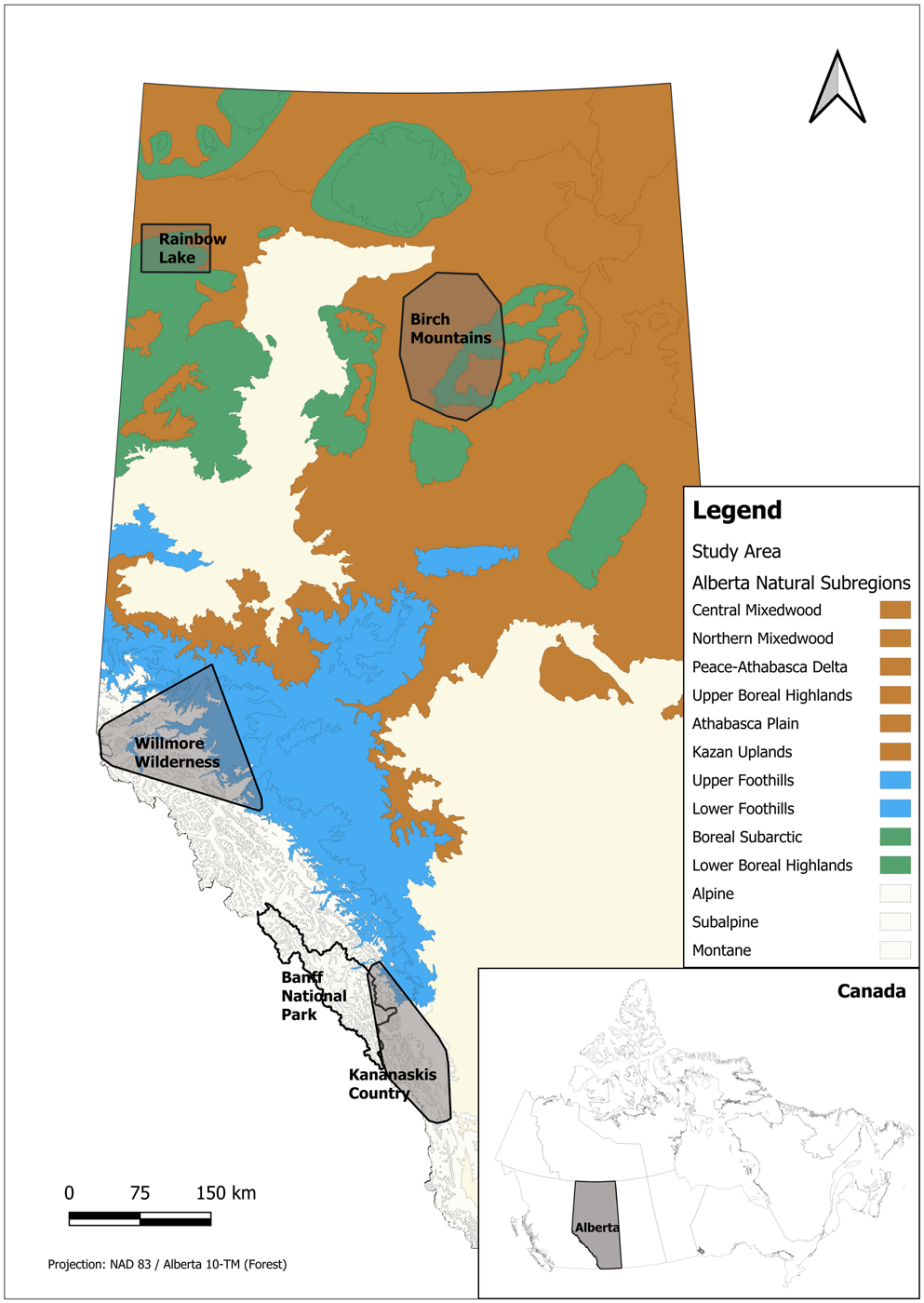
The province of Alberta (inset in Canada) is a varied landscape categorised into natural subregions (coloured alike to reflect combinations outlined in Table 3). Areas in pale yellow were considered wolverine nonhabitat. The areas for which wolverine density was estimated are Rainbow Lake, Birch Mountains, Willmore Wilderness, Banff National Park and Kananaskis Country.

All available datasets that included serial repeat detections of wolverines across the province of Alberta were compiled. We assessed whether data collection met required assumptions of spatially explicit capture–recapture (SECR) models (Borchers and Efford 2008; Efford 2011; Efford, Dawson, and Borchers 2009; Royle et al. 2014) wherein detectors (or samplers) are deployed across a study area and detect animal occurrences serially across multiple occasions, for example, weeks or months. SECR models require that individuals are identifiable—typically by pelage markings or DNA analysis. The spatial extent of each animal's detection and the detection rate inform the estimation of model parameters: (1) *g*_*0*_, the probability of detecting the animal at the hypothetical geometric centre of its spatial range (also called ‘activity centre’); and (2) σ, the half‐normal rate of decay in detection moving away from that centre. Based on these estimates, activity centres for each animal are summed together across the sampling extent to estimate abundance, and divided by the estimation area to estimate density, *D*. Other classes of models can accommodate fully unmarked individuals to estimate density, such as spatial count (SC) models (Chandler and Royle 2013), random encounter and staying time (REST) models (Nakashima, Fukasawa, and Samejima 2018) and time in front of the camera (TIFC) models (Becker et al. 2022). These have been used on other species (Burgar et al. 2018; Doran‐Myers et al. 2021; Pettigrew, Sigouin, and St‐Laurent 2021; Sun et al. 2022) but demonstrate some issues (Fisher et al. 2023), so we used only datasets compatible with SECR models (Supporting Information). These were obtained from two regions in the Rocky Mountains; we also relied on two previously published density estimates from the boreal forest (Figure 1).

### 2.2 Sampling: Willmore Wilderness

**FIGURE 2:**
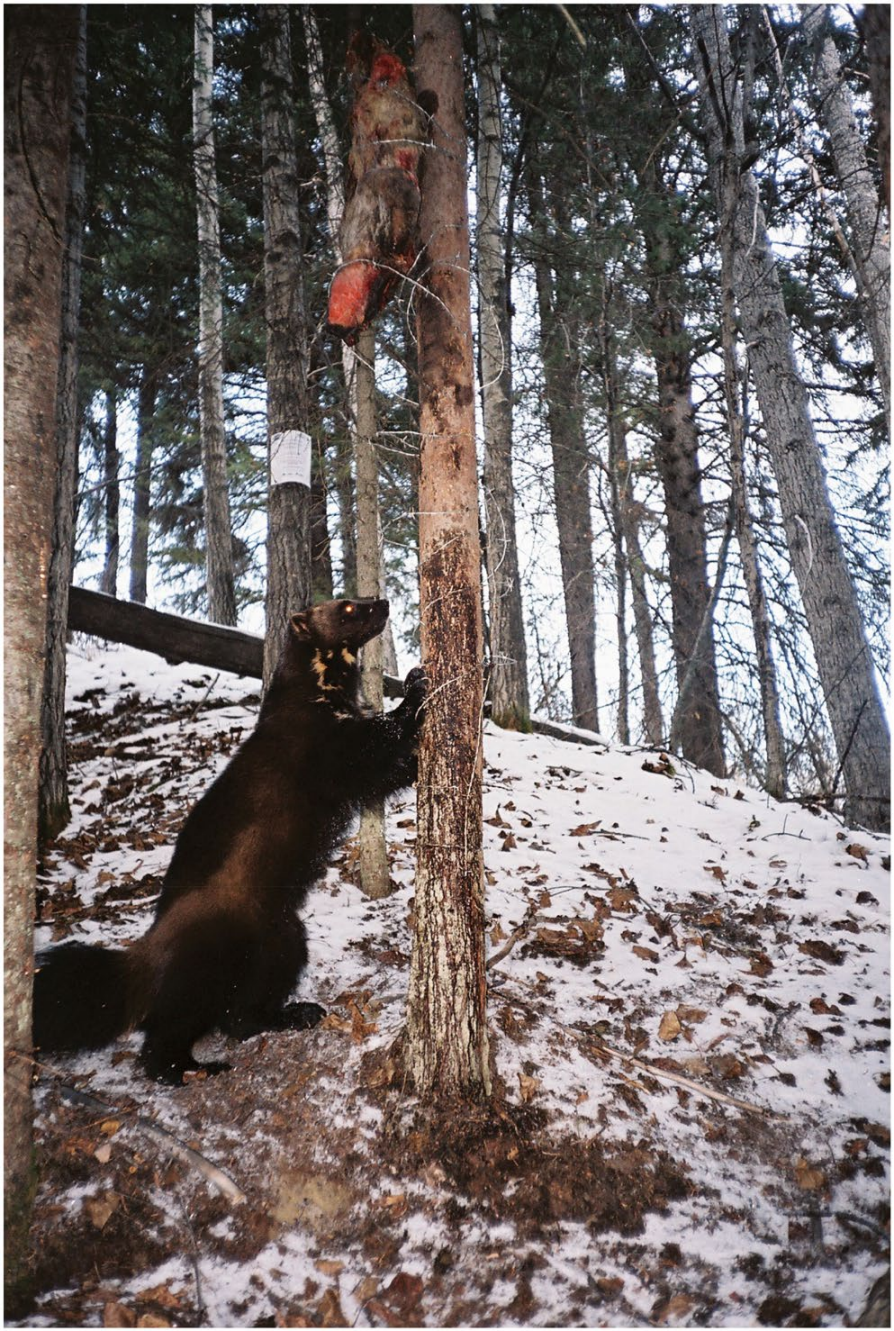
Wolverine ( *Gulo gulo* ) at a combined hair and camera trap sampling station in the Foothills of Alberta, Canada. Wolverines climbed the tree to get the beaver ( *Castor canadensis* ) bait and left hair samples on barbed wire, while also leaving a photographic record.

We used serial detection data from 66 wolverine sampling sites deployed in a systematic design of 12 × 12‐km^2^ grid cells across the Willmore Wilderness. At each site, a single Reconyx RM30 or PM30 infrared‐triggered digital camera faced the hair trap—a tree wrapped loosely with barbed wire, baited with a frozen beaver ( *Castor canadensis* ) carcass and scent lure (O'Gorman's, Montana, USA), as described in Fisher and Bradbury (2014) (Figure 2). Cameras were programmed at high sensitivity, five images per trigger, 1 s apart and rapid fire with no delay. Hairs and images were collected monthly (*t* = 3 surveys) January–March 2005–2006 and identified to individuals by Wildlife Genetics International (Nelson, British Columbia, Canada). Methods and data are reported by Fisher et al. (2013) wherein they informed an aspatial density estimate using a method (capture–recapture) that suffers from an *ad hoc* assignment of study area size (Royle et al. 2014).

### 2.3 Sampling: Kananaskis Country

We used serial detection data from 54 wolverine sampling sites deployed in a systematic design of 12 × 12‐km^2^ grid cells across Kananaskis Country. At each site, the same camera and hair trap combination was used as in the Willmore Wilderness, as described in Fisher and Bradbury (2014) (Figure 2). Cameras were programmed at high sensitivity, five images per trigger, 1 s apart and rapid fire with no delay. Hairs and images were collected monthly (*t* = 3 surveys) from January to April 2011 and identified to individuals by Rocky Mountain Research Station (Missoula, Montana) as reported in Heim et al. (2017).

### 2.4 Density Models: Willmore and Kananaskis

We used data in each region in separate SECR models (Efford 2004), in which habitat covariates can be used to estimate density across different habitat types (Efford and Mowat 2014; Efford and Fewster 2013). As wolverine occurrence has been shown to vary markedly with snow cover, anthropogenic disturbance, natural habitat and terrain ruggedness (Barrueto et al. 2022; Chow‐Fraser et al. 2022; Heim et al. 2017, 2019; Mowat et al. 2020; Stewart et al. 2016), we used those covariates in a density model with the intention of using these different habitat‐specific densities to extrapolate across mountain regions. We created competing models wherein density varied with: behaviour as a permanent learned response (b), variation by detector (k), a permanent detector‐specific learned response (bk), time (t) or a trend in those variables (B,K,T). We implemented the models using R version 4.3.2 (R Core Team, 2021). Maximum‐likelihood estimation methods were used to estimate the SECR model parameters of Wilmore and Kananaskis regions, and we used the R package *secr* (Efford 2024). R package *sf* (Pebesma and Bivand 2023; Pebesma 2018) was used to incorporate the spatial data.

### 2.5 Density: Rocky Mountain National Parks

We used an existing SECR density estimate and an extrapolated map created by Barrueto et al. (2022). Their 2018–2020 study spanned 4896 km^2^ of Banff National Park (Alberta) and Yoho and Kootenay National Parks (British Columbia). Their sampling protocol used a run‐pole and alligator clip grab trap to capture wolverine images (Reconyx PC 900, Holmen, WI) and hair samples (Magoun et al. 2011; Royle et al. 2011). Like Fisher et al. (2013), sampling occurred December–April and used the same scent lure, bait and sampling interval (~ 30 days). Hair was identified to species and individuals using the same protocols as in Fisher et al. (2013) (Wildlife Genetics International). Barrueto et al. (2022) sampled 202 occasions across the 3 years with up to four occasions per year (occasion duration mean = 30.8, SD = 5.5) (Barrueto et al. 2022). From 2018 to 2020, 21 individuals were detected at 28 (78%) of the 36 sampled sites, 17 of the 21 individuals (81%) were detected multiple times and 16 (76%) were detected at multiple sites. Remote cameras at the sites detected unidentified (no DNA or chest pattern obtained; Table 1) wolverine on 40 occasions.

**TABLE 1:** Maximum‐likelihood estimates from wolverine density models in the Willmore Wilderness, Alberta, in 2005–2006 including density (D), estimated standard deviation of density (SD), the activity centre detection probability (g0) and the detection rate of decay (*σ*). Models that failed to converge are shaded in grey.

Model	Density/1000 km^2^	SD	g0 (SD)	*σ* (in m), (SD)	AIC	ΔAIC
D ~ TRI + snow + HFI	0.18	0.24	5.60e‐2 (9.77e‐3)	1.80e4 (1.56e3)	602.37	0.00
D ~ snow + veg	5.43	2.66	5.20e‐2 (8.73e‐3)	1.78e4 (1.53e3)	602.76	0.39
D ~ TRI + veg + HFI	0.17	0.19	5.63e‐2 (9.71e‐3)	1.79e4 (1.49e3)	603.42	1.05
D ~ snow + veg + HFI	8.97	3.78	5.22e‐2 (8.82e‐3)	1.76e4 (1.49e3)	603.93	1.56
D ~ TRI + HFI	0.22	0.48	5.70e‐2 (9.90e‐3)	1.75e4 (1.46e3)	604	1.63
D ~ all	0.16	0.19	5.67e‐2 (9.83e‐3)	1.79e4 (1.52e3)	604.22	1.85
D ~ TRI + snow + veg	5.43	9.6	5.20e‐2 (8.73e‐3)	1.78e4 (1.54e3)	604.77	2.40
D ~ TRI + snow	0.28	0.44	5.40e‐2 (9.51e‐3)	1.83e4 (1.64e3)	605.19	2.82
D ~ snow	1.61	0.66	5.10e‐2 (8.63e‐3)	1.80e4 (1.54e3)	606.67	4.30
D ~ HFI	3.22	0.64	5.07e‐2 (8.61e‐3)	1.79e4 (1.51e3)	607.58	5.21
D ~ veg + HFI	5.68	2.66	5.18e‐2 (8.78e‐3)	1.78e4 (1.49e3)	608.51	6.14
D ~ snow + HFI	1.92	0.81	5.09e‐2 (8.63e‐3)	1.79e4 (1.53e3)	608.57	6.20
Null	2.57	0.53	5.24e‐2 (8.92e‐3)	1.78e4 (1.53e3)	609.55	7.18
D ~ TRI	2.93	1.27	5.20e‐2 (8.91e‐3)	1.79e4 (1.54e3)	611.43	9.06
D ~ veg	2.23	1.53	5.23e‐2 (8.91e‐3)	1.79e4 (1.54e3)	611.5	9.13
D ~ TRI + veg	5.40	22.6	5.18e‐2 (8.85e‐3)	1.79e4 (1.54e3)	613.35	11.00
g0 ~ Bk	2.60	0.55	3.44e‐2 (6.73e‐3)	2.01e4 (2.08e3)	871.58	269.00
g0 ~ bk	2.61	0.55	3.49e‐2 (6.93e‐3)	1.99e4 (2.03e3)	888.9	287.00
g0 ~ B	3.32	0.76	2.50e‐2 (6.37e‐3)	1.77e4 (1.51e3)	895.18	293.00
g0 ~ k	2.78	0.59	3.88e‐2 (7.74e‐3)	1.80e4 (1.53e3)	911.45	309.00
g0 ~ b	3.31	0.81	2.51e‐2 (7.92e‐3)	1.78e4 (1.50e3)	911.96	310.00
g0 ~ t	2.57	0.53	1.93e‐2 (8.28e‐3)	1.78e4 (1.53e3)	915.24	313.00
g0 ~ T	2.57	0.53	3.14e‐2 (7.85e‐3)	1.78e4 (1.53e3)	916.59	314.00

*Note:* Also listed are model selection results (AIC and ΔAIC). ‘TRI’ = Terrain ruggedness index, ‘snow’ = snow cover on the ground on April 15th (Landsat), ‘HFI’ = human footprint index (ABMI.ca) and ‘veg’ = vegetation cover (ABMI.ca). Trapping covariates on *g*_*0*_ are as follows: T = time factor of each occasion, T = linear trend over occasions, b = learned response after the first capture, B = Markovian response on captures, bk = site‐specific learned response, Bk = site‐specific Markovian response and k = changes in site effectiveness after any animals captured. Shaded lines represent models with convergence problems indicating an unsupported model.

Density estimates from SECR models had been extrapolated across the whole study area including parts of Alberta and the neighbouring province of British Columbia (Barrueto et al. 2022). To get an estimate for the Rocky Mountain National Parks within the boundaries of Alberta, we masked their spatial density layer to include only landscapes within the province of Alberta and extracted the mean density within these boundaries.

### 2.6 Density: Boreal Forest

Boreal wolverine densities were derived from two sources. In the Birch Mountains region of northcentral Alberta (Figure 1), combined camera and hair‐snag surveys on log‐cabin traps and run‐pole samplers (Magoun et al. 2011) were conducted in the winter of 2016–2017 (Alberta Conservation Association 2020; Webb et al. 2019, 2016). Wolverines were identified from camera images (rather than DNA) based on unique chest markings. In total, 31 sampling locations were surveyed across 1976 km^2^ for 140 single‐day occasions. There were 77 detections of 7 individuals (3F, 4 M). Applying SECR models with different covariates, ranked using AIC and validated against different buffer size assumptions, a most likely density of 1.4 wolverines per 1000 km^2^ (95% CI 0.66–3.00) was estimated. Full details are in the Alberta Conservation Association (2020).

The second study occurred in a 2268 km^2^ area of the Rainbow Lake region of northwestern Alberta and used similar methods to the Birch Mountains study, with the addition of live captures at log‐cabin traps, as described elsewhere (Scrafford et al. 2017, 2018; Scrafford and Boyce 2018; Webb et al. 2016). The Rainbow Lake study deployed sampling sites in a nonrandom design that balanced spacing (~10 km apart) and access routes. Sites were baited with beaver carcasses and sampled from November through April 2014–2015 and 2015–2016. Herein, detections were obtained using two methods: (1) live traps constructed to capture, tag and collar individuals, which could then be repeatedly captured; and (2) run poles with cameras (*sensu* Magoun et al. (2011)) were also used to detect wolverines and identify them through their unique pelage.

Scrafford et al. (2024) used SECR models (Efford 2004) to estimate density. Occasion length was set to 14 days. As live captures and camera detections were often not independent at a single live trap, Scrafford et al. (2024) combined these data as a single detection within each occasion. Density was modelled as a function of sex, session, detector type and behaviour (trap‐specific learned response), as well as spatial (landcover) covariates. The 2014 density estimate was 6.34 wolverines/1000 km^2^ (4.42–9.09) and the 2015 density estimate was 7.14 wolverines/1000 km^2^ (5.02–10.15). The average density estimate across sessions was 6.74 wolverines/1000 km^2^ (Scrafford et al. 2024). Adults were discerned from subadults based on evidence of lactation or mammary tissue (females) or descended testes (males), as well as tooth wear, from captured animals; added inference was derived from movement behaviour via GPS telemetry. Proportion of adults (estimate = 57% [44–70]) did not statistically differ from the proportion of subadults (estimate = 43% [30–56]) in Rainbow Lake (*p* = 0.300 [−0.241–0.838]) (Scrafford et al. 2024).

### 2.7 Extrapolating Density Estimates

To estimate the number of wolverines province‐wide, we needed to extrapolate the density estimates from study landscapes across larger areas. This is always a difficult endeavour (Foster and Harmsen 2012; Luo et al. 2020; Pettigrew, Sigouin, and St‐Laurent 2021; Williams, Nichols, and Conroy 2002). How well do the density estimates from one area reflect the entire region? Do estimates come from local hotspots of high density, or alternatively, local cold spots of low density? How do wolverine densities vary across space? Unfortunately, very little information exists to guide these decisions. Few studies have been able to examine how wolverine density changes across space, in relation to environment—such as topography, natural landcover and anthropogenic disturbance (but see Lofroth and Krebs (2007) and Barrueto et al. (2022)). However, more research exists on how wolverine *distribution* varies with these factors. There is substantial evidence that environmental factors affect wolverine distributions (and by extension and densities). Topographic ruggedness (elevational differences between adjacent areas) is a consistent predictor of distribution, as is coverage of snow and ice, persistent spring snow (snow on the ground as of April/May), conifer and broadleaf landcover and anthropogenic disturbance (Aubry et al. 2022; Barrueto et al. 2022; Barrueto, Sawaya, and Clevenger 2020; Chow‐Fraser et al. 2022; Fisher et al. 2013, 2022; Heim et al. 2017; Kortello, Hausleitner, and Mowat 2019; Scrafford et al. 2018; Stewart et al. 2016).

We had two major options to estimate density. First, we could use those habitat covariates identified as important in SECR models and use spatial data in a Geographic Information Systems (GIS) to map out predicted densities for each habitat polygon, and then sum them. We could also do this for each sex if habitat selection differed among sexes. This approach assumes that—for landscapes where density was modelled against landscape covariates—these relationships are stationary across the entire extrapolation landscape. Stationarity is hotly debated in model extrapolation (Bar‐Massada and Belmaker 2017; Miller 2012; Osborne, Foody, and Suárez‐Seoane 2007) and we suspected latent variables (such as harvest pressure) would also impact density. We chose not to use this method for these reasons. Second, we could assume that the density in the study areas captures the mean density across the surrounding landscape. This suffers the same problem with stationarity but assumes that the latent variables affecting density in the SECR estimate are represented and functioning in the extrapolation landscape. We chose this approach. Based on Tobler's first law of geography, we assumed study landscapes are likely closer in character to the larger landscapes in which they are embedded—in this case, landscapes defined by the Alberta Natural Regions and Subregions schema (NSR; Natural Regions Committee 2006). NSRs differ by climate, topography, precipitation and edaphics, generating different dominant landcovers and vegetation communities. As NSRs capture much of the climatic, topographic and vegetational heterogeneity associated with wolverine distribution, we chose to use these as a basis for extrapolating density estimates from study areas to the rest of the province.

## 3 Results

### 3.1 Willmore Wilderness

We detected 26 individuals at 59 of 66 sites and all three sampling occasions. Persistent spring snow and vegetation cover best explained wolverine density, estimated at 5.43 wolverines/1000 km^2^ (S.D. 2.66; ΔAIC = 6.79 from the null model; Table 1). Other models had lower AIC scores but displayed convergence problems, suggesting the maximum‐likelihood estimator selected a local minimum, and were disregarded (Harville 1977; Mantel and Myers 1971). None of the trapping and behaviour covariates explained wolverine density better than did the null model (Table 1). Adding human footprint index (HFI) or topographic ruggedness index (TRI) did not sufficiently account for Kullback–Leibler (K‐L) information loss in the model to warrant inclusion (Arnold 2010; Burnham and Anderson 2002).

### 3.2 Kananaskis Country

We detected five individuals at 8 of 54 sites and all three sampling occasions. The null model best explained wolverine density; addition of trapping or behaviour covariates did not explain sufficient K‐L information to overcome the parameter penalty (Table 2). Of the environmental covariates, again several models failed to properly converge (Table 2, shaded rows). Of those models that did converge, persistent spring snow had the lowest AIC score, but with ΔAIC = 1.21 from the null, did not sufficiently account for K‐L information loss in the model to warrant inclusion (Arnold 2010; Burnham and Anderson 2002). Likewise, behavioural and landscape covariates did not explain added variance. Therefore, the best‐supported, most parsimonious model (the null model) estimates wolverine density = 0.92 per 1000 km^2^.

**TABLE 2:** Maximum‐likelihood estimates from wolverine density models in Kananaskis Country, Alberta, in 2011–2012, including density estimate, standard deviation (SD) the activity centre detection probability (g0) and the detection rate of decay (*σ*). Models that failed to converge are shaded in grey.

Model	Density/1000 km^2^	SD	g0 (SD)	σ (in m.), (SD)	AIC	ΔAIC
D ~ snow	0.71	0.56	4.21e‐2 (2.08e‐2)	1.37e4 (3.24e3)	100.05	0.00
D ~ HFI	0.11	0.049	3.77e‐2 (1.97e‐2)	1.50e4 (3.96e3)	100.63	0.58
Null	0.92	0.50	3.47e‐2 (1.81e‐2)	1.53e4 (4.51e3)	101.26	1.21
D ~ TRI	0.75	0.57	3.76e‐2 (1.89e‐2)	1.44e4 (3.69e3)	101.38	1.33
D ~ HFI + snow	0.59	0.41	4.20e‐2 (2.11e‐2)	1.38e4 (3.34e3)	101.76	1.71
D ~ TRI + snow	0.66	0.66	4.25e‐2 (2.09e‐2)	1.36e4 (3.20e3)	101.96	1.91
D ~ veg	0.76	0.71	3.72e‐2 (1.92e‐2)	1.45e4 (3.79e3)	102.3	2.25
D ~ HFI + veg	0.08	0.042	3.76e‐2 (1.98e‐2)	1.50e4 (4.10e3)	102.62	2.57
D ~ TRI + HFI	0.15	0.079	3.79e‐2 (1.99e‐2)	1.49e4 (4.06e3)	102.63	2.58
D ~ veg + TRI	0.26	1.55	3.35e‐2 (1.93e‐2)	1.54e4 (5.26e3)	102.88	2.83
D ~ snow + TRI + HFI	0.30	0.26	4.27e‐2 (2.13e‐2)	1.38e4 (3.39e3)	103.37	3.32
D ~ veg + TRI + HFI	0.09	0.17	3.86e‐2 (1.96e‐2)	1.46e4 (3.84e3)	104.35	4.3.0
g0 ~ bk	0.99	0.76	1.61e‐2 (1.25e‐2)	1.99e4 (1.31e4)	126.93	26.900
g0 ~ Bk	0.97	0.60	2.17e‐2 (1.38e‐2)	1.77e4 (7.56e3)	128.22	28.20
g0 ~ k	1.02	0.64	2.12e‐2 (1.38e‐2)	1.72e4 (6.69e3)	132.04	32.00
g0 ~ T	0.92	0.50	6.24e‐2 (3.70e‐2)	1.52e4 (4.47e3)	132.2	32.20
g0 ~ B	1.51	1.33	1.53e‐2 (1.43e‐2)	1.49e4 (4.05e3)	132.54	32.50
go ~ t	0.92	0.50	4.36e‐2 (3.13e‐2)	1.53e4 (4.49e3)	133.15	33.10
g0 ~ b	1.04	0.87	2.71e‐2 (2.96e‐2)	1.52e4 (4.45e3)	134.92	34.90

*Note:* ‘TRI’ = terrain ruggedness index, ‘snow’ = snow cover on the ground on April 15th (Landsat), ‘HFI’ = human footprint index (ABMI.ca) and ‘veg’ = vegetation cover (ABMI.ca). Trapping covariates on *g*_*0*_ are as follows: T = time factor of each occasion, T = linear trend over occasions, b = learned response after the first capture, B = Markovian response on captures, bk = site‐specific learned response, Bk = site‐specific Markovian response and k = changes in site effectiveness after any animals captured. Shaded lines represent models with convergence problems indicating an unsupported model.

### 3.3 Density Extrapolation for Alberta

We adopted the following extrapolations based on Alberta Natural Subregions' characteristics (Natural Regions Committee 2006) most closely shared with those study areas from which density estimates were derived (Table 3):

1. The Willmore Wilderness is reflective of protected, undeveloped Northern Rocky Mountain habitat, and its density estimate should be extrapolated within Willmore Wilderness and Kakwa Wilderness Parks. This includes alpine, subalpine and montane Natural Subregions.
2. Kananaskis Country is a mix of various land‐use zones ranging from fully protected to recreational to those allowing (extensive) resource extraction. It is reflective of climatic, topographic and anthropogenic conditions typically of the eastern slopes and its density estimate was extrapolated across the upper foothills and lower foothills of Natural Subregions.
3. The Canadian Rocky Mountain Parks estimate is reflective of Southern Rocky Mountain landscapes where recreation and transportation are the only anthropogenic activities. Barrueto et al. (2022) and Mowat et al. (2020) had already estimated density for Banff and Waterton National Parks and the adjacent mountain regions, then extrapolated to Jasper National Park. The density estimate for these combined areas includes alpine, subalpine and montane Natural Subregions.
4. The Birch Mountains study area is a mix of upland mixedwood (i.e., conifer and deciduous) characteristic of the lower boreal plain, with moderate spring snow and some anthropogenic landscape development. We contend that this density estimate should be extrapolated to the Athabasca Plain, Canadian Shield/Kazan uplands, Peace–Athabasca Delta, upper boreal highlands Natural Subregion, central mixedwood and northern mixedwood.
5. The Rainbow Lake area is characterised by lowland spruce forest with some mixedwood and lowland hygric bogs. It has a high energy development footprint. Here, we extrapolate this density estimate to the lower boreal highlands Natural Subregion and boreal subarctic Natural Subregion.
6. The Grassland Natural Region and Parkland Natural Region are no longer inhabited by wolverines. Little is known about wolverines in the dry mixedwood (boreal) Natural Subregion of Alberta. However, as this subregion has a (relatively) warmer climate, is dominated by aspen forests and is 50%–70% cultivated—all negatively associated with wolverine occurrence—, we have inferential evidence that wolverines do not currently inhabit the dry mixedwood Natural Subregion.

**TABLE 3:** Estimated density of wolverines extrapolated across regions in Alberta, Canada.

Region	Region area (km^2^)	Summed Natural Subregion(s) Area (km^2^)	Density estimate (1000 km^−2^)	# Gulo	Lower 95% CI	Upper 95% CI
Jasper National Park	11,228	11,228	4.31	48	10.22	114.53
Banff National Park	6641	6641	4.35	29	4.38	58.24
Waterton Natl. Park	505	505	1.56	1	0.26	1.80
Willmore Wilderness	4568	5217	5.43	28	1.15	55.51
Kakwa Wilderness	649					
Kananaskis Country/Upper Foothills	21,537	66,436	0.92	61	0.00	125.56
Lower Foothills	44,899					
Athabasca Plain	13,525	238,006	1.40	333	157.08	714.02
Shield/Kazan Uplands	9719					
Peace–Athabasca Delta	5535					
Upper Boreal Highlands	11,858					
Central Mixedwood	167,856					
Northern Mixedwood	29,513					
Lower Boreal Highlands	55,615	67,438	6.74	455	261.66	683.82
Boreal Subarctic	11,823					
Total	395,471	395,471	3.46	955	434.75	1753.48

*Note:* Shaded adjacent rows illustrate regions grouped together for extrapolation based on shared environmental characteristics. For example, density estimated for the Willmore Wilderness is also extrapolated to adjacent Kakwa Wilderness, and the summed area for the two is reported in that column, multiplied to obtain a total number of animals for those areas.

Following these criteria, we estimated that there were 955 wolverines (95% CI = 435, 1753) in the province of Alberta (Table 3) at the time of these surveys.

## 4 Discussion

Density is the basis for harvest management decisions as well as conservation status under IUCN criteria, so is a vital parameter for informed conservation. We estimated 955 wolverines in Alberta (95% CI 435–1753) from existing data. However, the Willmore Wilderness estimate is now almost two decades old and the Kananaskis estimate is almost a decade old. Research in the adjacent National Parks and the British Columbia landbase to the west shows a substantial decline in population density over one decade (Barrueto et al. 2022; Barrueto, Sawaya, and Clevenger 2020). Newer estimates are needed for Alberta's provincial mountain landbase. The boreal estimates are valuable in that they are newer, and boreal wolverines have been relatively less studied than mountain wolverines (save for the extensive telemetry work in the northwest (Scrafford et al. 2017; Scrafford et al. 2018; Scrafford and Boyce 2018)).

We note the substantial variability in density estimates from these studies. The Willmore sample size was reasonable (26 individuals detected at the majority of sites), but Kananaskis Country sample size was very small—only five individuals were detected by DNA, at 14% of study sites. All estimates fall within the range observed elsewhere in the literature, so are plausible; however, there is always the possibility of bias for various reasons (Efford 2004; Efford, Borchers, and Byrom 2009; Royle et al. 2014; Zimmermann, Foresti, and Rovero 2016). Assuming estimates reflect biological reality, the widely different estimates from different areas suggest a high degree of heterogeneity in wolverine habitat suitability across Alberta, and (or) differing population pressures such as harvest. Much more research is needed to parse these possibilities apart.

Identifying the environmental factors that affect and explain density within and among study areas is a central challenge. Trapping records can sometimes provide information on relative densities, and these have been used to infer changes in wolverine populations through time (Brodie and Post 2010). However, trapping records are highly sensitive to effort, itself variable with pelt prices and other factors, such that scientific confidence in these data is often fairly low (DeVink, Berezanski, and Imrie 2011; Jung et al. 2020; McKelvey et al. 2011). Moreover, because of Alberta's quota system (1 per registered fur management area) and the differences in accessibility and area among traplines, the relationships between wolverine density and trapper take may be artificially truncated. Alternatively, species associations can sometimes index a species' density. European lynx ( *Lynx lynx* ) have been suggested as closely tracking wolverine numbers in Scandinavia (Andrén et al. 2011; Khalil, Pasanen‐Mortensen, and Elmhagen 2014; Mattisson et al. 2011). However, an explicit analysis of this hypothesis suggests this relationship does not hold for Nearctic wolverines and the smaller, hare‐specialised Canada lynx ( *Lynx canadensis* ; Chow‐Fraser et al. 2022). In summary, there is little hard evidence that harvest data—for lynx or wolverines—are a solid basis for extrapolating wolverine densities. Extrapolation based on landscape characteristics thus appears a reasonable expedient.

In Alberta, mountain landscapes are comparatively well studied. The boreal forest remains our biggest information gap. We have two density estimates from landscapes that are very small (1%–3% of their respective subregions), and no information at all from the heaviest‐developed landscapes in the Alberta Oil Sands Region, where ongoing mammal monitoring has detected very few wolverines (ABMI.ca; ACMELab.ca). Given these caveats, we estimate fewer than 1000 total wolverines in Alberta. IUCN status assessments are based on the number of adult animals in the population: In the only live‐capture study in Alberta, Scrafford et al. (2024) observed a ratio of 57% adults to 43% subadults in the northwestern boreal forest. If these ratios hold for the greater population, then there are fewer than 600 (544) wolverine adults in Alberta, with broad confidence intervals. This ratio is more conservative (leaning towards adults) than other estimates, which range from 25% adults in Alaska (Dalerum, Shults, and Kunkel 2008) to 37% adults (males)/43% adults (females) in the Yukon (Kukka et al. 2017)—both estimates derived from harvested carcasses.

A quick but illustrative analysis suggests our estimate is robust to assumptions about how to extrapolate wolverine densities to Natural Subregions. If instead fully half the landbase of the boreal (152,722 km^2^) were assigned the higher density estimate (6.7/1000 km^2^) and the other half of the boreal were assigned the lower estimate (1.4/1000 km^2^)—a scenario that defies known wolverine responses to landcover and anthropogenic development—, there would still be an estimated < 750 mature individuals in the province. This number has implications for wolverine conservation status: Alberta has adopted the IUCN Red List criteria for species assessments, one of which is the number of extant mature individuals https://www.iucnredlist.org/resources/categories‐and‐criteria and this number falls below the threshold for a listed designation.

Notably, however, IUCN criteria also include trend information—whether the population is increasing, decreasing or stable. Here, repeated surveys of established study sites are needed. Only one population trend analysis based on robust, repeatable sampling at a large spatial scale has been produced in Canada (Barrueto et al. 2022); many more are needed. A coordinated, provincial‐scale monitoring programme conducted over time would reveal a great deal about wolverine populations and trends, as illustrated by the exemplary work of Bischof et al. (2020).

## 5 Conclusions

The majority of research on wolverine density in Alberta has been planned and executed by Universities, National Parks, private biologists and NGOs to meet their own priorities. Without a provincial monitoring programme such as Bischof et al. (2020), density research has been spotty around the province. A spatially and temporally extensive wolverine monitoring programme is required to understand wolverine density in this key part of wolverines' range. Building on known effective designs (Alberta Conservation Association 2020; Fisher et al. 2013; Heim et al. 2017) and integrating with efforts in the National Parks and British Columbia (Barrueto et al. 2022; Barrueto, Sawaya, and Clevenger 2020; Mowat et al. 2020) and to the south in the United States (Carroll et al. 2021; Lukacs et al. 2020) would provide more power to such a programme. Stratifying the province according to factors known to affect wolverine density—anthropogenic development, vegetation cover and spring snow—and then sampling large landscapes within them (min. 10,000 km^2^, given large home ranges 200–1000 km^2^ in size) would provide much more robust data on wolverine densities in the province. Finally, this programme should be repeated several times within a decade so we can understand trends in density. The declines in density in the most protected areas of western Canada over the last decade (Barrueto et al. 2022) do not bode well for wolverines dealing with the many working landscapes in Alberta, where development has been occurring at a rapid pace for the last few decades. Some areas, such as Kananaskis, may be subject to extirpation without source populations from adjacent mountain National Parks. Our estimated provincial adult population falls well below 1000—one of the IUCN thresholds for a listed species, and we strongly recommend a reassessment of wolverine status and considering commensurate conservation actions.

## Author Contributions

**Jason T. Fisher:** conceptualization (lead), investigation (equal), project administration (lead), resources (equal), supervision (lead), writing – original draft (equal), writing – review and editing (equal). **Mehnaz Jahid:** data curation (lead), formal analysis (lead), investigation (lead), methodology (lead), software (lead), validation (lead), writing – original draft (equal), writing – review and editing (equal). **Robin Gutsell:** conceptualization (equal), data curation (equal), funding acquisition (lead), project administration (equal), resources (equal), supervision (equal), writing – review and editing (equal). **Anne Hubbs:** conceptualization (equal), data curation (equal), funding acquisition (equal), project administration (equal), supervision (equal), writing – review and editing (equal). **Laura L. E. Cowen:** formal analysis (supporting), methodology (equal), supervision (supporting), validation (supporting), writing – review and editing (supporting). **Mirjam Barrueto:** data curation (equal), investigation (equal), writing – review and editing (supporting). **Nicole Heim:** data curation (equal), investigation (supporting), writing – review and editing (equal). **John Paczkowski:** data curation (equal), investigation (supporting), writing – review and editing (equal).

## Conflicts of Interest

The authors declare no conflicts of interest.

## Data Availability

All code and data are publicly available at https://github.com/Mehnaz‐Jahid/Alberta_wolverine_density.
